# Human prion disease with a G114V mutation and epidemiological studies in a Chinese family: a case series

**DOI:** 10.1186/1752-1947-2-331

**Published:** 2008-10-17

**Authors:** Jing Ye, Jun Han, Qi Shi, Bao-Yun Zhang, Gui-Rong Wang, Chan Tian, Chen Gao, Jian-Min Chen, Cun-Jiang Li, Zheng Liu, Xian-Zhang Li, Lai-Zhong Zhang, Xiao-Ping Dong

**Affiliations:** 1 Department of Neurology, Xuan-Wu Hospital, Capital University of Medical Science, Beijing 100053, PR China; 2 State Key Laboratory for Infectious Disease Prevention and Control, National Institute for Viral Disease Control and Prevention, Chinese Center for Disease Control and Prevention, Ying-Xin Rd, Beijing 100052, PR China; 3 Affiliated Hospital of Jining Medical College, Shandong 272029, PR China

## Abstract

**Introduction:**

Transmissible spongiform encephalopathies are a group of neurodegenerative diseases of humans and animals. Genetic Creutzfeldt-Jakob diseases, in which mutations in the *PRNP *gene predispose to disease by causing the expression of abnormal PrP protein, include familial Creutzfeldt-Jakob disease, Gerstmann-Straussler-Scheinker syndrome and fatal familial insomnia.

**Case presentation:**

A 47-year-old Han-Chinese woman was hospitalized with a 2-year history of progressive dementia, tiredness, lethargy and mild difficulty in falling asleep. On neurological examination, there was severe apathy, spontaneous myoclonus of the lower limbs, generalized hyperreflexia and bilateral Babinski signs. A missense mutation (T to G) was identified at the position of nt 341 in one *PRNP *allele, leading to a change from glycine (Gly) to valine (Val) at codon 114. PK-resistant PrP^Sc ^was detected in brain tissues by Western blotting and immunohistochemical assays. Information on pedigree was collected notably by interviews with family members. A further four suspected patients in five consecutive generations of the family have been identified. One of them was hospitalized for progressive memory impairment at the age of 32. On examination, he had impairment of memory, calculation and comprehension, mild ataxia of the limbs, tremor and a left Babinski sign. He is still alive.

**Conclusion:**

This family with G114V inherited prion disease is the first to be described in China and represents the second family worldwide in which this mutation has been identified. Three other suspected cases have been retrospectively identified in this family, and a further case with suggestive clinical manifestations has been shown by gene sequencing to have the causal mutation.

## Introduction

Transmissible spongiform encephalopathies (TSEs) are a group of neurodegenerative diseases of the central nervous system (CNS). The best known of human forms of TSE, Creutzfeldt-Jakob diseases (CJD), are classified into three subtypes, sporadic CJD (sCJD), iatrogenic CJD (iCJD), and genetic or familial CJD (gCJD or fCJD) [1]. Hereditary forms of human TSE in which mutations in the prion protein gene (*PRNP*) predispose to disease include fCJD, Gerstmann-Sträussler-Scheinker syndrome (GSS) and fatal familial insomnia (FFI) [2].

To date, about 55 mutations associated with or directly linked to human TSEs have been identified [3]. Here we report a Chinese family with a mutation at codon 114 (G114V) of the *PRNP *gene. The index case had clinical features, electroencephalogram (EEG) and magnetic resonance imaging (MRI) findings similar to sporadic CJD. We also present data on four suspected cases of fCJD in five consecutive generations of the family.

## Case presentation

### Clinical features

**Figure 1:**
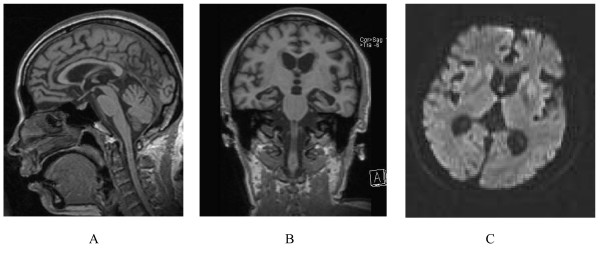
**(A and B) Magnetic resonance imaging, showing clear bilateral atrophy of cortex, brainstem and cerebellum. **(C) Diffusion weighted imaging, displaying high signal in the caudate nucleus and putamen.

A 47-year-old Han-Chinese woman was hospitalized with a 2-year history of progressive dementia, tiredness, lethargy and mild difficulty in falling asleep. The initial complaint was tiredness and loss of sleep. Several months after the onset, she developed difficulty in communication and was unable to work. This was followed by a gradually progressive dementia and emotional lability. The family described increased appetite, and complex visual hallucinations. About 17 months after onset, the patient was first hospitalized and CSF 14-3-3 was negative at that time. On this admission, the patient was bedridden. On neurological examination there was severe apathy, spontaneous myoclonus of the lower limbs, generalized hyperreflexia and bilateral Babinski signs. An EEG displayed slow waves at 5 to 6 Hz, which were marked bilaterally in the frontal lobes and precentral regions. MRI of the brain showed bilateral atrophy of the cerebellar cortex, brainstem and cerebellum (Figure 1A and 1B). On diffusion weighted imaging (DWI), there were high signals in the caudate nucleus, the putamen and the periventricular regions (Figure 1C). Biochemistry of the cerebrospinal fluid (CSF) was normal; however, CSF 14-3-3 protein was not performed. One week later, the patient was discharged [from hospital and she died at home about 75 days later at the age of 47.

### Epidemiologic data

**Figure 2:**
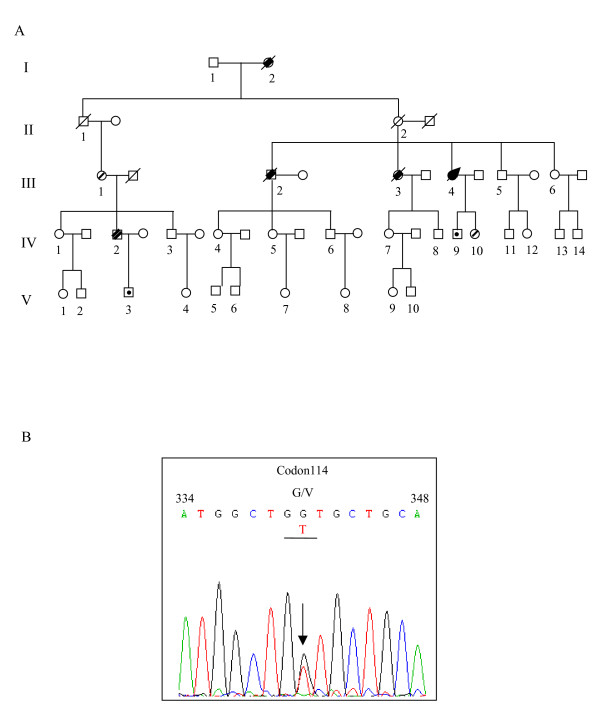
**(A) Pedigree of the G114V inherited prion disease family.** Affected patients are described in the text. Open square, male; open circle, female; filled square with arrow, proband case; square or circle with prolonged diagonal lines, deceased cases; square or circle with overstriking double diagonal lines, patients with neurologic signs according to medical records; square or circle with fine line, asymptomatic carriers with the G114V mutation; square or circle with dot, persons having been confirmed not carrying the G114V mutation. (B) *PRNP *sequencing showed a point mutation on one allele at position 114. The arrow indicates the position where both G and T were present.

Information on pedigree was obtained by interviews with family members. A total of 49 family members (including spouses) were retrospectively or directly investigated (Figure 2A). Thirty-three of the family members belonged to the proband's mother's lineage and 14 belonged to her uncle's (her mother's brother) lineage. The proband's maternal grandmother was said to have died with similar clinical symptoms. The proband's elder brother developed neurological symptoms at the age of 45 years and died 1 year after the onset. Her elder sister presented with similar clinical manifestations at age of 35 years and died 2 years after onset. The son of her first cousin (IV 2) had limited intellectual ability from childhood and discontinued his education at grade 4 of elementary school. He was hospitalized for investigation of progressive memory impairment 2 years ago at the age of 32 years. On examination, he had impairment of memory, calculation and comprehension, mild ataxia of the limbs, tremor and a left Babinski sign. He is still alive.

### PRNP analysis

Brain autopsy of the proband was performed shortly after death with informed consent. Genomic DNA was extracted from the brain using Qiagen's DNA purification kit according to the manufacturer's instructions. The *PRNP *open reading frame was amplified by polymerase chain reaction (PCR) using a protocol and primers described elsewhere [4]. The genotype at codon 129 of *PRNP *was determined by digestion with the restriction endonuclease Nsp I. Analysis of *PRNP *sequences was performed by direct sequencing in a MacBAC sequencer (Pharmacia, USA). A missense mutation (T to G) was identified at the position of nt 341 in one *PRNP *allele, leading to change from glycine (Gly) to valine (Val) at codon 114 (Figure 2B). No other nucleotide exchange was found in the rest of the *PRNP *sequence. Nsp I digestion and direct sequencing of the amplified product revealed a methionine homozygous genotype at codon 129 of *PRNP*. To identify the distribution of this point-mutation in the family, blood samples of five other family members, including the son of her first cousin (IV 2), were collected and the *PRNP *genes were sequenced. As suspected, the same G114V mutation was observed in the *PRNP *gene of IV 2. In addition, two other health family members, the proband's daughter (IV 10, age of 22) and the mother of the second case (III 1, age of 61), were found to have the same missense mutation. The son of the proband case (IV 9, age of 24) and the son of IV 2 (V 3, age of 9) were confirmed to have a wild-type *PRNP *sequence without such mutation. All tested family members were homozygous for methionine (M/M) at codon 129 of *PRNP *as in the profile of Han Chinese [5].

### Proteinase K (PK)-resistant PrP assays

**Figure 3:**
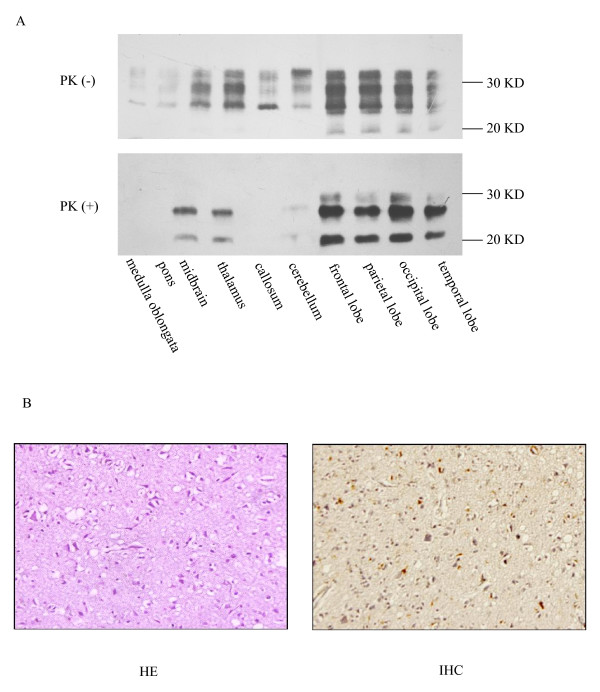
**(A) Western blotting analysis of brain tissue from the proband.** Brain samples are treated with Proteinase K (+) at a concentration of 50 μg/ml or without Proteinase K (-) before electrophoresis. (Top) Western blot of brain homogenates without Proteinase K digestion. (Bottom) Western blot graph of brain homogenates with Proteinase K digestion. Each brain region is indicated at the bottom of the image. Molecular weight standards are shown on the right. (B). Neuropathological assays of occipital lobe. Hematoxylin and eosin staining (left) and immunohistochemistry with monoclonal antibody 3F4 (right) (×400).

Western blotting was performed to identify the presence of PrP^Sc ^in the brain tissue of the patient. The brain tissue sample was homogenized in 9 volumes of lysis buffer (100 mM NaCl, 10 mM EDTA, 0.5% Nonidet P-40, 0.5% sodium deoxycholate, 10 mM Tris, pH 7.5) according to the protocol described elsewhere [6]. An aliquot of the homogenate from cerebrum was incubated with PK (at a final concentration at 50 μg/ml) at 37°C for 1 hour. Three PK-resistant PrP^Sc ^bands ranging from *Mr *21 to 27 kDa were detected with predominance of monoglycosylated PrP^Sc ^indicating type 1 PrP^Sc ^(Figure 3A). To examine the distribution of PrP^Sc ^in different brain regions, aliquots of 10% tissue homogenates were prepared from various brain regions and analyzed by Western blot. PK-resistant PrP was detected in the midbrain, thalamus, cerebellum, frontal lobe, temporal lobe, parietal lobe and occipital lobe, but not in the medulla oblongata, pons or corpus callosum. These findings seemed to be closely related to the level of the total PrP signal before PK-digestion in each homogenate (Figure 3A). The electrophoretic pattern of PrP^Sc ^was the same in all preparations, with predominance of monoglycosylated PrP^Sc^.

### Histological and immunohistochemical (IHC) assays

Paraffin sections of occipital lobe (5 μm in thickness) were subjected to conventional staining with hematoxylin and eosin (HE) and severe and extensive vacuolation was identified in the tested tissues (Figure 3B). To identify PrP^Sc ^in brain tissues, slices of occipital lobe were immunostained using a protocol described elsewhere [7]. Briefly, the slices were treated with 4 M guanidine hydrochloride (GdnHCl) at 4°C for 90 minutes, followed by microwave irradiation in distilled water for 25 minutes. The slices were exposed to the PrP-specific monoclonal antibody 3F4 at a dilution of 1:500 overnight at 4°C. For visualization of immunostaining, the slices were developed with a commercial ready-to-use system (Beijing Zhongshan Golden Bridge Biotechnology, China). The slices were counterstained with hematoxylin, dehydrated, and mounted in glycerolvinyl alcohol. Positive PrP^Sc ^immunoblots were found in many of the tested tissues, especially in the region of the gray matter. The deposits of PrP^Sc ^were restricted mostly to the neuronal cytoplasm. No obvious PrP^Sc ^deposits were observed in extracellular areas (Figure 3B).

## Conclusion

This family with G114V inherited prion disease is the first to be described in China and represents the second family worldwide in which this mutation has been identified. The patient presented with clinical features similar to sporadic CJD, including a progressive neuropsychiatric disturbance, dementia, myoclonus and pyramidal signs. Cerebellar signs were observed relatively later, but became marked. MRI revealed findings consistent with those often seen in sporadic CJD, but the EEG did not show the typical periodic complexes of sporadic CJD. The CSF 14-3-3 was negative 1 year after onset. Typical spongiform degeneration and PrP^Sc ^deposits were observed in the brain and Type-I PrP^Sc ^was detected in various brain regions. Three other suspected cases have been retrospectively identified in this family, and a further case with suggestive clinical manifestations has been shown to have the causal mutation by gene sequencing. The age at clinical onset in this pedigree ranges from 32 to 45 years, which is somewhat later than cases in a Uruguayan family [3], which was the first to be described with a G114V mutation. However, the duration of illness and other clinical manifestations are quite similar in the two families. Interestingly, in our pedigree, the mother (III 1) of the second case carries the G114V mutation in her *PRNP *gene and remains healthy at the age of 61 years. This suggests that some other unknown factors may influence the phenotype of genetic human TSE.

## Competing interests

The authors declare that they have no competing interests.

## Authors' contributions

JY, JH and QS contributed equally to this article, in which JY identified the proband clinically, JH was responsible for the epidemiological study and QS was responsible for the laboratory assays. BYZ, GRW and CT performed the neuropathological assays. CG and JMC performed the genetic tests. CJL, ZL, XZL and LZZ collected and analyzed the clinical data. XPD organized the study and was the major contributor in writing the manuscript.

## Consent

Written informed consent was obtained from the patient for publication of this case report and any accompanying images. A copy of the written consent is available for review by the Editor-in-Chief of this journal.
